# Inhibition of ITGA2 suppresses cervical tumorigenesis and metastasis by targeting the AKT/mTOR signaling pathway

**DOI:** 10.1016/j.gendis.2024.101328

**Published:** 2024-05-18

**Authors:** Haixia Luo, Jingjing Chang, Yiting Ren, Xiu Zhang, Yuanxing Li, Miao Huo, Hongyuan Wang, Xin Yang, Jianbing Liu, Qingmei Zhang, Yueyang Zhao, Wei Wang

**Affiliations:** a Department of Obstetrics and Gynecology, The Second Hospital of Shanxi Medical University, Taiyuan, Shanxi 030001, China; b Pathology Department, School of Medicine, Stanford University, California 94305, USA; c School of Basic Medical Sciences, Shanxi Medical University, Taiyuan, Shanxi 030001, China; d School of Applied Science, Taiyuan University of Science and Technology, Taiyuan, Shanxi 030024, China

Cervical cancer (CCa) is a substantial global health concern, and its lymph node metastasis (LNM) significantly diminishes patients' survival rates.^1^ Hence, a thorough investigation into the molecular mechanisms driving this progression is vital. Integrin alpha 2 (ITGA2) plays critical roles in various tumorigenic processes via cancer-related signaling pathways.^2^ In this study, we advanced the understanding of ITGA2's influence on CCa by revealing its role in promoting CCa LNM. Mechanistically, ITGA2 up-regulates SNAIL, instigating epithelial–mesenchymal transition (EMT) and activating the protein kinase B (AKT)/mammalian target of rapamycin (mTOR) pathway, thereby augmenting CCa LNM. Remarkably, the inhibitor E7820, by down-regulating ITGA2 expression, shows promise in attenuating EMT and LNM of CCa. Therefore, E7820 could be developed as a potential therapeutic agent for the treatment of CCa LNM.

In our previous study,^3^ we noted elevated ITGA2 expression in CCa compared with non-tumor tissue using microarrays (Fig. S1A–C). Analysis of the CCa database via Kaplan–Meier plotter revealed that ITGA2 mRNAs predicted poor overall survival in CCa (Fig. 1A). To investigate the role of ITGA2 in CCa, we modulated ITGA2 expression in SiHa cells, a well-established HPV16-positive cervical cancer cell line that is consistent with CCa tissues we sequenced. ITGA2 knockdown inhibited SiHa cell proliferation, increased apoptosis, and caused G1 phase arrest. Conversely, ITGA2 overexpression enhanced cell viability, reduced apoptosis, and led to S phase accumulation (Fig. S1D–J). Transwell assays confirmed ITGA2's significant role in modulating cell migration and invasion. Knockdown of ITGA2 substantially hindered these processes, while conversely, ITGA2 overexpression significantly amplified cell migration and invasion (Fig. 1B, C). These consistent findings were further supported by wound healing assays, underscoring ITGA2's impact (Fig. S2A, B).

**Figure 1:**
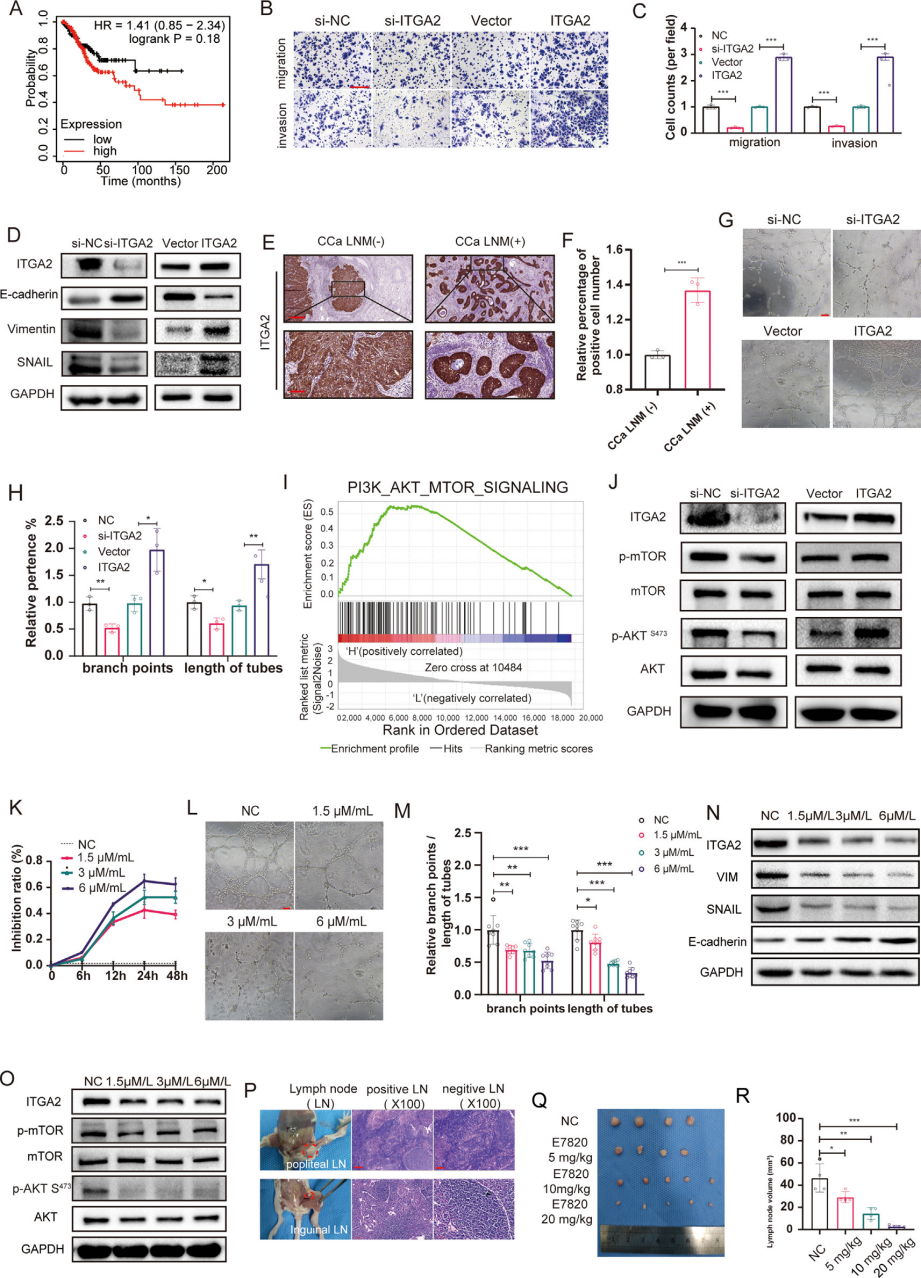
The expression and regulation of ITGA2 in CCa. **(A)** Kaplan–Meier survival analysis of CCa patients with high ITGA2 versus those with low ITGA2. **(B)** Transwell assays were performed to investigate the effects of ITGA2 on the invasion abilities of indicated cells. Scale bar, 200 μm. **(C)** Statistical analysis of transwell assays. **(D)** Western blot analysis of EMT markers in indicated cells with knockdown or overexpression of ITGA2. **(E)** Representative images of ITGA2 immunohistochemistry staining in CCa without LNM [LNM (−)] and with LNM [LNM (+)]. **(F)** The relative percentage of ITGA2 positive cell number in CCa tissues. (**G**)The effects of ITGA2 on the tube formation of HLECs ( × 200). Scale bar, 100 μm. **(H)** Statistical analysis of tube formation of HLECs. **(I)** Gene sets enriched by high ITGA2 versus low ITGA2 through gene set enrichment analysis. **(J)** Western blotting of key molecules in the AKT/mTOR signaling pathway. Overexpression of ITGA2 enhances the expression of p-AKT and p-mTOR. **(K)** E7820 inhibited the proliferation of SiHa cells determined by CCK8 assay. **(L)** The effects of E7820 on the tube formation of HLECs ( × 200). Scale bar, 100 μm. **(M)** Statistical analysis of tube formation of HLECs. **(N)** Western blot analysis of EMT markers in indicated cells treated with E7820. **(O)** Western blot analysis of key molecules in the AKT/mTOR signaling pathway in indicated cells treated with E7820. **(P)** Representative pictures of hematoxylin-eosin staining of metastasis positive and negative lymph nodes in different parts of mice. **(Q)** Representative images of popliteal lymph nodes. **(R)** Histogram analysis of the volume of lymph nodes. Error bars represent the mean ± standard deviation of three independent experiments. ∗*P* < 0.05, ∗∗*P* < 0.01, ∗∗∗*P* < 0.001. CCa, cervical cancer; ITGA2, integrin alpha 2; EMT, epithelial–mesenchymal transition; LNM, lymph node metastasis; HLECs, human lymphatic endothelial cells; AKT, protein kinase B; mTOR, mammalian target of rapamycin.

Given ITGA2's effect on cell migration and invasion,^4^ we explored its potential to initiate EMT in CCa. In our set of CCa tissue samples, we investigated the correlation between ITGA2 expression and markers associated with EMT. The results distinctly revealed a positive association between ITGA2 levels and EMT markers, such as vimentin (*r* = 0.5034, *P* < 0.01), along with EMT-inducing transcription factors like SNAIL (*r* = 0.3414, *P* < 0.05). Intriguingly, an inverse relationship was observed with the epithelial marker E-cadherin (*r* = −0.4363, *P* < 0.01) (Fig. S2C–F). Subsequently, ITGA2 knockdown significantly up-regulated E-cadherin expression while down-regulating SNAIL and vimentin in CCa cells, overexpression of ITGA2 resulted in the opposite effect (Fig. 1D).

LNM is the most common metastatic site and is intricately linked with EMT.^5^ Hence, we embarked on a detailed exploration to assess the correlation between ITGA2 and the occurrence of LNM. Immunohistochemistry on CCa (with LNM and without LNM) specimens corroborated significant ITGA2 overexpression in CCa tissues with LNM compared with CCa tissues without LNM (Fig. 1E, F). Following that, we investigated ITGA2's influence on tube formation by human lymphatic endothelial cells (HLECs), a crucial step in cancer LNM. Notably, culture supernatants from ITGA2-knockdown cells substantially reduced HLEC tube formation, highlighting a significant inhibitory effect. Conversely, ITGA2 overexpression exerted a promotive influence, augmenting HLEC tube formation (Fig. 1G, H).

In investigating ITGA2-mediated migration in CCa, we categorized cases based on ITGA2 mRNA levels (high and low) and conducted gene set enrichment analysis. The analysis showed a significant association between high ITGA2 expression and the AKT pathway (Fig. 1I). Notably, Figure 1J illustrates that silencing ITGA2 could cause a discernible inhibition in AKT phosphorylation, while ITGA2 overexpression could markedly augment this phosphorylation. Additionally, focusing on mTOR, a direct downstream target of the AKT pathway, we observed a commensurate alteration in phosphorylation levels following the modulation of AKT pathway activity, regulated by ITGA2 expression levels.

Given our finding that ITGA2 plays a significant role in LNM, we employed E7820, an inhibitor of ITGA2, to evaluate its potential for treating LNM in CCa (Fig. S2G). Determining the half maximal inhibitory concentrations (IC50) of E7820 via MTT and live/dead cell staining revealed that E7820 significantly reduced the viability of SiHa cells, with IC50 values of 1.4 mM, without causing harm to normal cells (Fig. S2H–J and Table S1). To determine optimal conditions, cells were treated with varying E7820 concentrations and incubation times. E7820 inhibited cell proliferation (1–5 μmol/mL) without inducing cell death (Fig. S2K). After 24 h, ITGA2 mRNA and protein significantly decreased (Fig. S2L, M). Thus, we chose 1.5 μmol/mL, 3 μmol/mL, and 6 μmol/mL for further study.

Subsequently, colony formation, CCK-8, and EdU assays were conducted to determine the effects of E7820 on cell proliferation. As depicted in Figure 1K and Figure S2N–P, 3A and B, E7820 inhibited the proliferation of SiHa cells as the concentration and exposure time increased. According to Figure S3C and D, the percentage of total apoptotic cells, including the early apoptotic portion and the late apoptotic portion, increased with the E7820 concentrations. Similarly, E7820 significantly increased the ratio of TUNEL-positive cells (Fig. S3E, F). According to the cell cycle analysis, E7820 significantly increased the number of cells in the G1 phase (Fig. S3G, H).

We observed that E7820 dose-dependently reduced the migration speed of SiHa cells (Fig. S3I, J) and the invasive and migratory abilities (Fig. S3K, L). Subsequently, HLEC tube formation revealed that E7820 inhibited HLEC tube formation in a dose-dependent manner (Fig. 1L, M). We also found that E7820 inhibited EMT via the AKT/mTOR signaling. Vimentin and SNAIL were dose-dependently reduced in E7820-exposed cells after 24 h, while E-cadherin expression increased (Fig. 1N). Immunofluorescence staining further confirmed these findings (Fig. S4A–H). Figure 1O shows that E7820 could inhibit the phosphorylation of Akt (Ser 473) and mTOR in SiHa cells in a dose-dependent manner. Considering these outcomes, E7820 appeared to effectively inhibit CCa cell invasion, migration, LNM, and EMT, primarily via the AKT/mTOR pathway.

To assess E7820's *in vivo* efficacy, we utilized subcutaneous xenograft and lymphatic metastatic models in female BALB/c nude mice (Fig. S4I). E7820 exhibited a dose-dependent inhibitory effect on tumor growth (Fig. S4J, K) and diminished Ki67-stained cells (Fig. S4L). Post-injection of tumor cells into the foot pads of nude mice, we removed and analyzed their popliteal and inguinal lymph nodes after 25 days (Fig. 1P). Notably, the volume of popliteal lymph nodes in the E7820 group was significantly smaller compared with the control group, indicating a substantial reduction in LNM rates (Fig. 1Q, R). These findings underscore E7820's potential in suppressing cervical cancer tumor growth and LNM *in vivo*.

In summary, our study links ITGA2 up-regulation to clinical and functional relevance in CCa. Inhibiting ITGA2 with E7820 suppresses EMT, lymphangiogenesis, and cell proliferation, suggesting a promising therapeutic target for CCa with LNM (Fig. S5).

## Ethics declaration

All experimental procedures were approved by the Medical Ethics Committee of The Second Hospital of Shanxi Medical University (No. 2019YX195) and performed in accordance with the relevant guidelines and regulations. Written informed consents were obtained from all patients.

## Author contributions

W.W. designed and supervised this project. H.L., J.C., and Y.R. analyzed the data and wrote the manuscript. H.L. and J.C. performed the experiments. J.L., Q.Z., Y.Z., and W.W., revised the manuscript. H.L. and Y.L. contributed to data interpretation. All authors read and approved the final manuscript.

## Conflict of interests

The authors declare no conflict of interests.

## Funding

This work is supported by grants from the Applied Basic Research Program of Shanxi, China (No. 20210302123277), the Key Research and Development Program of Shanxi, China (No. 201903D321152), and the National Natural Science Foundation of China (No. 82373107).
