# Supplementary material for: Inhibition of ITGA2 suppresses cervical tumorigenesis and metastasis by targeting the AKT/mTOR signaling pathway
Source: Genes Dis. 2024 May 18;12(2):101328. doi: 10.1016/j.gendis.2024.101328 (PMC11616036; doi:10.1016/j.gendis.2024.101328)
Supplement: Multimedia component 1 [file mmc1.docx]

**Supplementary Figure Legends**

**Figure S1** Comprehensive experimental analysis revealing ITGA2 and functional markers in CCa. **A** Heatmap of differentially expressed mRNAs in the three comparison groups. **B and C** Expression of ITGA2 is upregulated in CCa. **D** The growth curves of cells transfected with indicated vectors were evaluated by CCK8 assays. **E** Representative images of colony formation. **F** Statistical analysis of colony numbers. **G** Representative images of flow cytometry analysis on cell apoptosis rate of indicated SiHa cells. **H** Statistical analysis of cell apoptosis. **I** Representative images of the cell cycle analysis of indicated SiHa cells. **J** Statistical analysis of cell cycle. **K** Representative images of the Edu of indicated SiHa cells. Scale bar, 1000 μm. **L** Representative images of the TUNEL of indicated SiHa cells. Scale bar, 1000 μm. **M** Statistical analysis of Edu assays. **N** Statistical analysis of TUNEL assays. All experiments were performed three times and data are presented as mean ± SD. *P < 0.05, **P < 0.01, ***P < 0.001, n.s, nonsignificant.

**Figure S2** Comprehensive analysis of ITGA2 modulation and E7820 inhibition in CCa cellular dynamics and proliferation **A** Wound healing assays show that motility is enhanced in ITGA2 overexpression cells, whereas ITGA2 knockdown suppresses motility in SiHa cells. Scale bar, 100 μm. **B** Statistical analysis of wound healing assays. **C** Representative images of ITGA2, E-cadherins, Vimentin and SNAIL IHC staining in CCa. **D- F** Pearson correlation analysis of ITGA2 and E-cadherins, Vimentin, or SNAIL expression in 15 CCa. CCa: cervical cancer; **G** Chemical structure of E7820. **H-I** Representative images of Calcein-AM/PI staining. **J** Statistical analysis of Calcein-AM/PI staining. **K** Real-time proliferation of SiHa cells following treatment with E7820. **L** Time course of ITGA2 mRNA after E7820 treatment at the indicated concentrations. **M** Time course of ITGA2 protein after E7820 treatment at the indicated concentrations. **N** E7820 inhibited the proliferation of Siha cells determined by CCK8 assay. **O** Representative images of cell colonies after treatment with various concentrations of E7820 for 24 h. **P** Statistical analysis of cell colonies assays. All experiments were performed three times and data are presented as mean ± SD. *P < 0.05, **P < 0.01, ***P < 0.001, n.s, nonsignificant.

**Figure S3** E7820 treatment modulates proliferation, apoptosis, and invasion in CCa. **A** Edu assays were conducted in cells after treatment with various concentrations of E7820 for 24 h. Scale bar, 1000 μm. **B** Statistical analysis of Edu assays. **C** Representative images of flow cytometry analysis on cell apoptosis rate of indicated SiHa cells. **D** Statistical analysis of cell apoptosis. **E** TUNEL assays were conducted in cells after treatment with various concentrations of E7820 for 24 h. Scale bar, 1000 μm. **F** Statistical analysis of TUNEL assays. **G** Representative images of the cell cycle analysis of indicated SiHa cells. **H** Statistical analysis of cell cycle. **I** Representative images of wound healing analysis on cell apoptosis rate of indicated SiHa cells. Scale bar, 100 μm. **J** Statistical analysis of wound healing assays. **K** Transwell assays were performed to investigate the effects of E7820 on the invasion abilities of indicated cells. Scale bar, 200 μm. **L** Statistical analysis of transwell assays. All experiments were performed three times and data are presented as mean ± SD. *P < 0.05, **P < 0.01, ***P < 0.001, n.s, nonsignificant.

**Figure S4** E7820 modulates EMT-associated protein expression and tumor growth in CCa **A-D** Immunoblotting analysis of EMT-associated proteins in indicated cells treated with E7820. **E-H** Statistical analysis of immunoblotting analysis. **I** Schematic representation of the establishment of the xenograft model. **J** Representative images of gross appearance of subcutaneous tumors from nude mice treated with saline solution and E7820. **K** Subcutaneous tumor growth curves of mice in different treatment groups. **L** Representative images of Ki67 IHC staining different treatment groups. **(M)** The relative percentage of ITGA2 positive cell number in xenograft tumor tissues. Error bars represent the mean ± S.D. of three independent experiments. *P < 0.05, **P < 0.01, ***P < 0.001.

**Figure S5**. Inhibition of ITGA2 Suppresses Cervical Tumorigenesis and Metastasis by Targeting the AKT/mTOR Signaling Pathway

**Supplementary Materials and Methods**

**Clinical specimens**

CCa tissues, HSIL tissues and normal cervical tissues were collected from patients who underwent gynecological surgery or colposcopy at the Second Hospital of Shanxi Medical University (Taiyuan, China) between 2019 and 2020. Patients in this study had not received radiotherapy or chemotherapy prior to surgery. All patients underwent radical hysterectomy and lymphadenectomy or cervical conization. Normal cervical tissues were obtained from patients undergoing hysterectomy under nonmalignant circumstances. The tissues were first immediately frozen in liquid nitrogen and then frozen at -80°C until RNA could be extracted. This study was approved by the Ethical Review Committee of the Second Hospital of Shanxi Medical University. The Declaration of Helsinki was followed in all patient studies.

**Chemicals**

The integrin inhibitor E7820 was purchased from Med Chem Express (NJ, USA). Antibodies against ITGA2, Vimentin, SNAIL + SLUG and E‐cadherin were obtained from Abcam. AKT and phospho-AKT were purchased from ABclonal Technology (Wuhan, China). The mTOR and phospho-mTOR antibodies were obtained from CST. GAPDH, rabbit anti‐rabbit IgG‐HRP and mouse anti‐rabbit IgG‐HRP were purchased from Santa Cruz Biotechnology (CA, USA).

**Cell culture**

SiHa and HaCaT cells (ATCC, Rockville, MD, USA) were cultured in DMEM (General Electric Company. USA) supplemented with 10% fetal bovine serum (Gibco; Thermo Fisher Scientific, Inc., Waltham, MA, USA), 100 mg/mL streptomycin, and 100 U/mL penicillin in a humidified atmosphere of 5% CO_2_ and 37°C.

**Transfection**

For transfection, we used siRNAs that target ITGA2 designed specifically by GenePharma (Suzhou, China) (Table S1). SiHa cells were plated in 6-well plates for 24 h and transfected with 30 pmol siRNA or scrambled control (GenePharma, Shanghai, China) using Lipofectamine 3000 (Invitrogen, MA, USA) according to the manufacturer's protocol. The ITGA2 overexpression plasmid was synthesized by Public Protein/Plasmid Library (Nanjing, China) and then transfected into cells using Lipofectamine 3000 and selected with puromycin (3 mg/ml, Sigma‒Aldrich, USA) for 7 days.

**qRT‒PCR and RT‒PCR**

qRT‒PCR and RT‒PCR were performed as described previously ^18^. The expression of the endogenous housekeeping gene GAPDH was used to calculate the relative expression level of genes using the 2^‐ΔΔCt^ method. The sequences of the primers used in qRT‐PCR are listed in Supplementary Table S2.

**In vitro cytotoxicity assay**

The in vitro cytotoxicity of E7820 was measured by a Cell Counting Kit-8 (CCK-8) (Meilunbio, China). Briefly, 5×10^3^ cells per well were plated in 96-well plates and treated with different concentrations of E7820 dissolved in DMSO for 24 h. The vehicle control was DMSO (6 μmol/mL). Then, the medium with E7820 or DMSO was replaced with 180 μL of fresh medium along with 10 μL CCK-8 solution in each well and incubated at 37 °C for 2 h. The absorbance of each well was determined by a microplate reader (SpectraMax M5, MD, USA) at a 450 nm wavelength. Growth inhibition rates were calculated with the following equation: inhibition ratio = (OD DMSO-OD drug) / (OD DMSO-OD blank) × 100%.

**Cell proliferation assay**

The rate of cell proliferation was assessed in real time using an xCELLigence RTCA dual-plate instrument (ACEA Biosciences, San Diego, CA, USA). Briefly, SiHa cells (6000 cells/well) were seeded into 100 µL of DMEM with 10% FBS in E-plates (ACEA Biosciences). The impedance value, expressed as the cell index, was recorded every 15 min. Cells were treated with the indicated concentration of E7820 or 6 μmol/mL DMSO in growth medium when they reached log phase, and impedance was recorded for another 96 h. The cell proliferation rate was derived from the slope of the line between two given time points. A cloning assay was performed on SiHa cells to determine their cloning capability. The Cell-Light™ EdU DNA Cell Proliferation Kit (Beyotime, Beijing, China) and CCK-8 (Meilunbio, Dalian, China) were also performed according to the manufacturer's instructions.

**Cell cycle and apoptosis assays**

Cell cycle analysis was conducted with propidium iodide (PI) staining by flow cytometry (Beckman-Coulter, Hialeah, FL) and analyzed using Modfit software. The Alexa Fluor® 488 Annexin V/Dead Cell Apoptosis Kit (Thermo Fisher Scientific) and TUNEL Apoptosis Assay Kit (Beyotime, Beijing, China) were used to identify apoptotic and dead cells.

**Human lymphatic endothelial cells (HLEC) tube formation assay**

HLECs were seeded into 24-well plates (precoated with Matrigel) and incubated for 6 hours. The lymphatic tubes were photographed using a Leica DMI8 microscope and quantified by the ImageJ Angiogenesis Analysis plugin.

**Transwell assay**

Transwell 24-well chambers with 8.0 μm pore sizes (Corning, USA) were used for cell migration and invasion assays. The cells dispersed in 200 μL serum-free medium (2 × 10^4^/well) were seeded into the upper chamber, and 600 μL medium containing 10% FBS was added to the lower section. The upper compartment was coated with Matrigel (BD Bioscience, USA) for the invasion assay or not for the migration assay. The cells that invaded or migrated the lower chamber at 6 h or 36 h, respectively, were stained with 0.1% crystal violet and counted. Each experiment was conducted independently in triplicate.

**Wound healing assay**

Cells were cultured on six-well plates for 48 h to reach 90-95% confluency and subjected to starvation in 1% FBS for 24 h. To inflict a wound, the cell monolayer was scratched using a sterile 10 μl pipette tip. After washing with PBS, the wells were filled with serum‐free culture medium. The extent of wound closure was assessed and photographed after 24 h under a Leica inverted microscope.

**Live/dead cell staining**

The live–dead cell staining kit (BestBio, Shanghai, China) was used according to the manufacturer’s instructions. Specifically, cells were cultured on 24-well plates at a density of 5 × 10^4^. PI and calcium-AM were diluted to final concentrations of 5 and 2 μM in staining reagent dilution buffer, respectively. After incubating with the indicated concentration of E7820 or 6 μmol/mL DMSO for 24 h, 200 μl of mixed solution was added to each specimen, and the cells were stained at 37 °C for 15 min.

**Immunocytochemistry**

Cells were seeded on sterile coverslips at a density of 1× 10^4^ overnight attachment. Following 24 hours of culture in the indicated concentration of E7820 or 6 μmol/mL DMSO, the coverslips were washed and fixed for 15 minutes with 4% paraformaldehyde and blocked in PBS containing 5% bovine serum albumin (BSA). Then, the coverslips were incubated with anti-vimentin antibody, anti-E‐cadherin antibody and anti-SNAIL + SLUG antibody (1:100) overnight at 4°C. Secondary anti-rabbit Alexa Fluor 594- and anti-mouse Alexa Fluor 488-labeled antibodies (Boster, Wuhan, China) were then applied for one hour, followed by nuclear counterstaining with DAPI for 15 min. Images were captured using a fluorescence microscope (Olympus Optical).

**Western blot analysis**

For western blot analysis, total proteins were extracted and electrophoresed by 12% SDS‒PAGE followed by loading on PVDF membranes (Millipore, Bedford, MA, USA). After blocking in 5% BSA, the membranes were exposed to primary antibodies, including anti-ITGA2, anti-E-cadherin, anti-vimentin, anti-SNAIL, anti-AKT, anti-p-AKT, anti-mTOR, anti-p-mTOR, and anti-GAPDH.

**Immunohistochemistry (IHC)**

IHC staining was performed to detect the expression of ITGA2, SNAIL, E-cadherin and vimentin in mouse tissues and human tissues. The antibodies were as described above for western blotting. IHC kits (Maxim, China) matching the species of the primary antibody were then used on the sections. Image-Pro Plus software quantified the integrated optical density of the IHC images.

**Hematoxylin-eosin (HE) staining**

The tissues were immersed in 4% paraformaldehyde, embedded in paraffin, and cut into 4 mm thick transverse sections. Hematoxylin and eosin were then applied to the sections.

**Animal experiments**

Female BALB/c nude mice at 4-5 weeks of age were purchased from GemPharmatech Co., Ltd. and housed in a specific pathogen-free facility. Mice were subcutaneously inoculated with 2×10^6^ SiHa cells suspended in 50% Matrigel Matrix (Corning, USA). After injection, tumor size was measured by using an external caliper. For the xenograft LNM model, 1 × 10^7^ cells were inoculated into the footpads of the mice. The lymph node volumes were calculated using the following formula: volume (mm^3^) = (length [mm]) × (width [mm])^2^ × 0.52. The footpad tumors and popliteal lymph nodes were removed for HE staining. For E7820 administration, the mice were randomized into 4 groups (n = 5) that were treated intraperitoneally with either 20 mg/kg E7820, 10 mg/kg E7820, 5 mg/kg E7820 or saline solution (NC) thereafter. Administration of vehicle or agents and measurement of tumor growth with a digital caliper were performed once every 5 days. Tumor volumes were calculated by the two-dimensional sizes of each tumor with the above formula. At the end of the experiment, the mice were weighed and sacrificed, and the tumors were weighed and dissected. The animal experimental protocols were approved by the Animal Ethics Committee of The Second Hospital of Shanxi Medical University.

**Statistical analyses**

Statistical analyses were performed using SPSS 13.0 and Prism 5.0 software. The independent sample t test was used to compare the difference between two groups. Kaplan-Meier method was used to analyze the relationship between the expression of ITGA2 and the overall survival time (OS) in patients with CCa. *P* < 0.05 was considered to be statistically significant.

| Supplementary Table 1. Sequences of siRNAs used in this study | |
| --- | --- |
| **siRNA sequences (5’-3’)** | |
| si-ITGA2 | GUGGUUGUGUGUGAUGAAUTT |
| si-NC | UUCUCCGAACGUGUCACGUTT |

Supplementary Table 2. Primer sequences used in qRT-PCR and PCR analysis

| **mRNA name** | **Forward Primer (5’-3’)** | **Reverse Primer (5’-3’)** |
| --- | --- | --- |
| ITGA2 | GGGAATCAGTATTACACAACGGG | CCACAACATCTATGAGGGAAGGG |
| GAPDH | CAGGAGGCATTGCTGATGAT | GAAGGCTGGGGCTCATTT |
