# Supplementary figures and images for: Inhibition of ITGA2 suppresses cervical tumorigenesis and metastasis by targeting the AKT/mTOR signaling pathway
Source: Genes Dis. 2024 May 18;12(2):101328. doi: 10.1016/j.gendis.2024.101328 (PMC11616036; doi:10.1016/j.gendis.2024.101328)

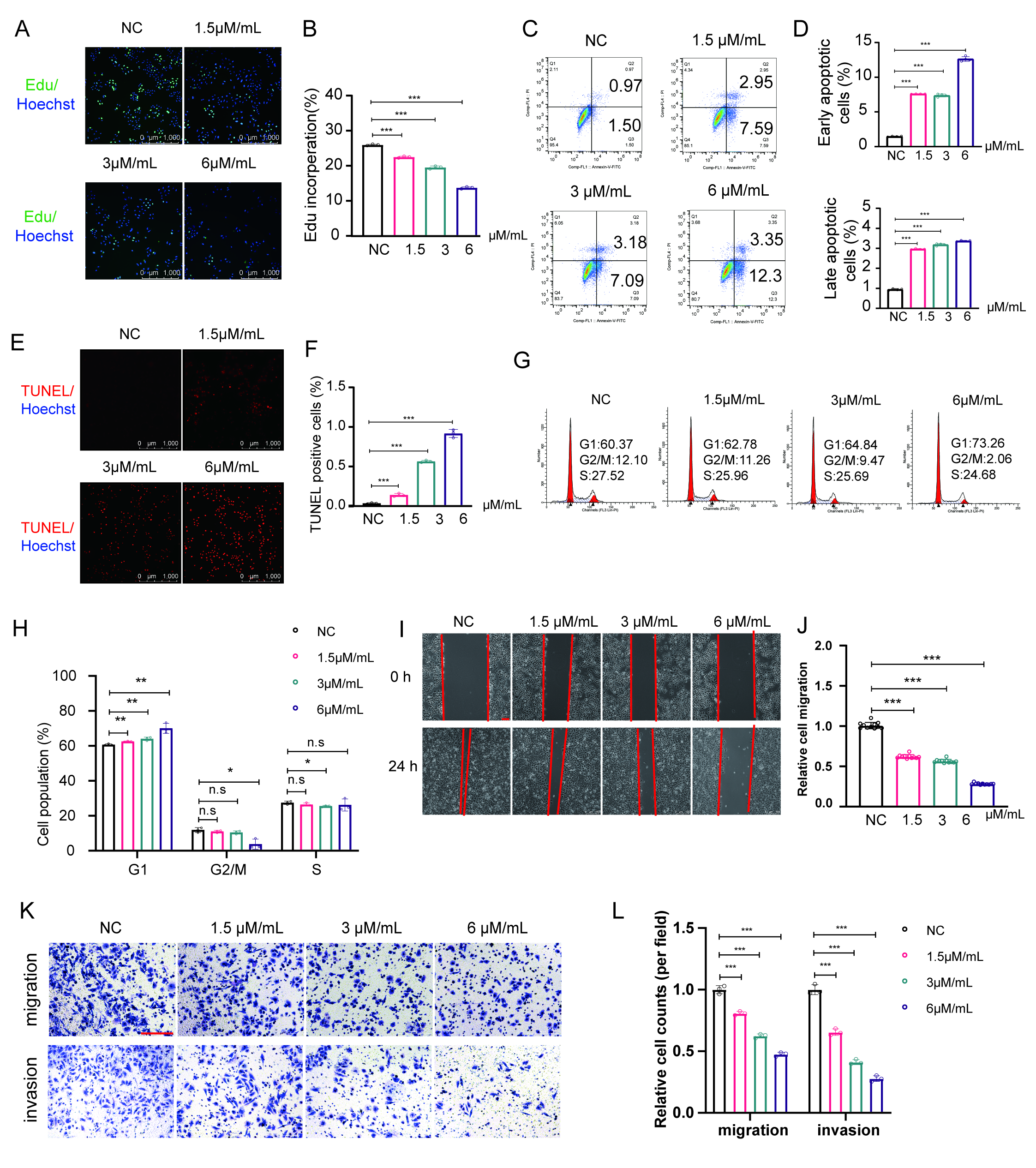

Supplement: Multimedia component 2 [file mmc2.zip › 5-29s3.tif]

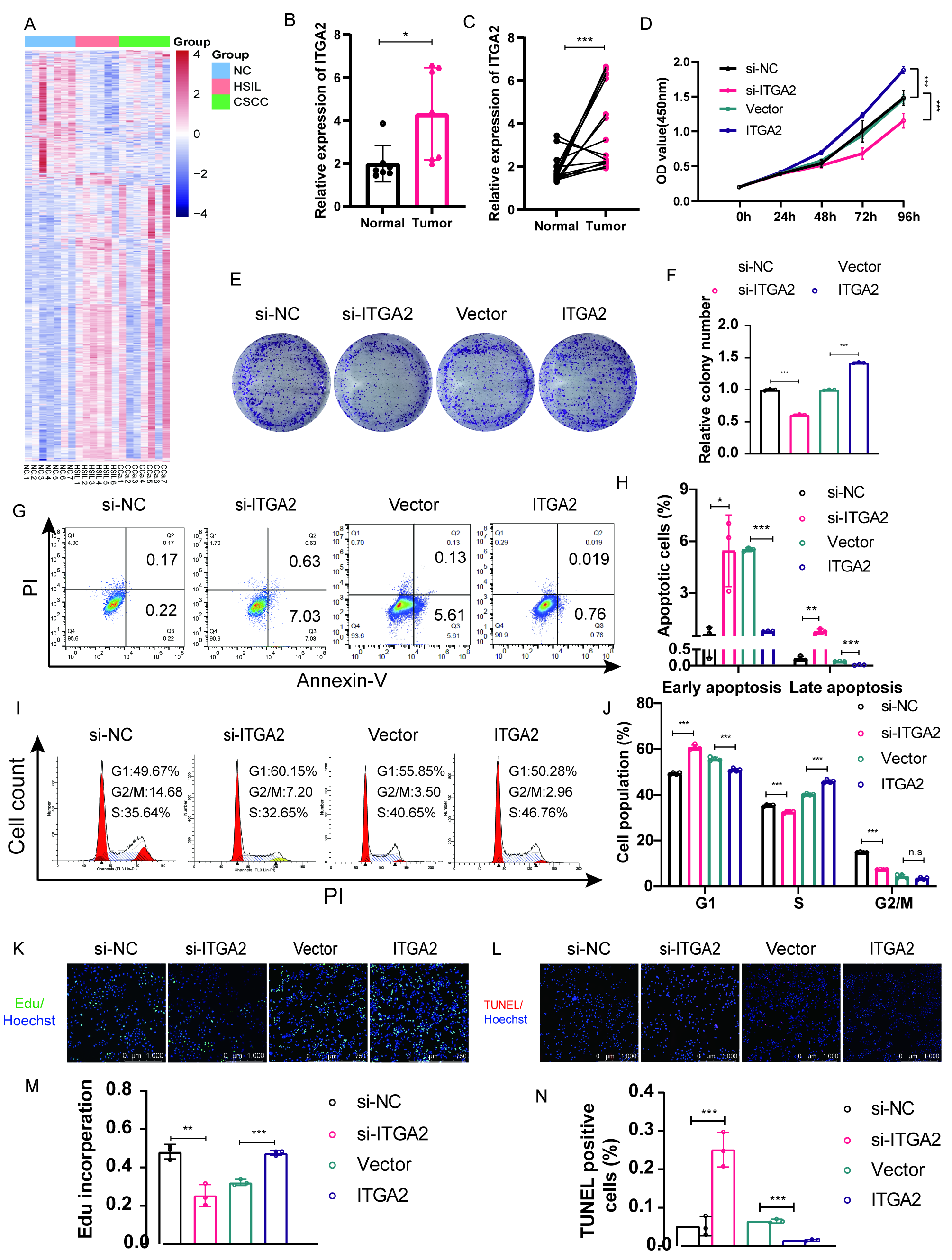

Supplement: Multimedia component 2 [file mmc2.zip › S1.tif]

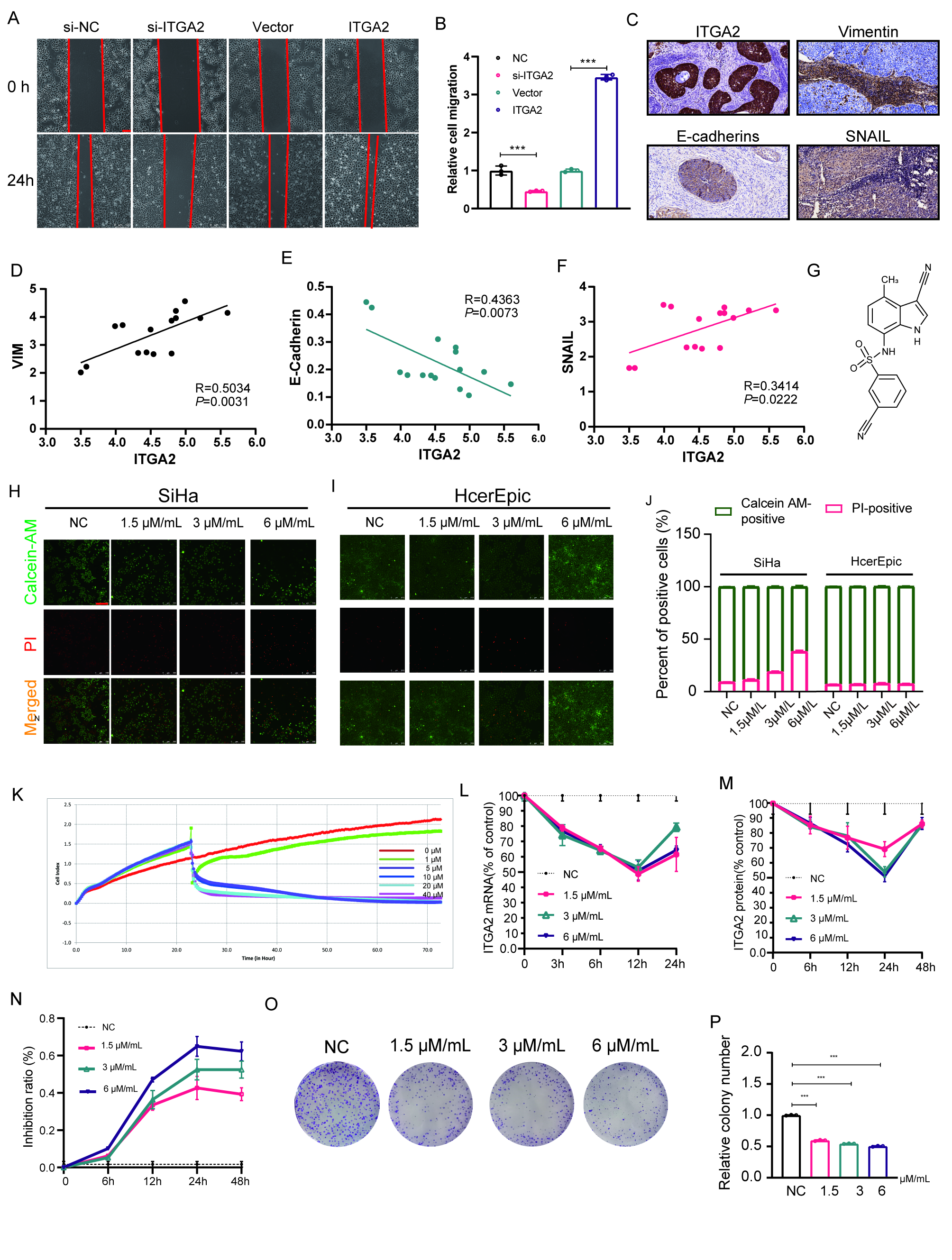

Supplement: Multimedia component 2 [file mmc2.zip › S2.tif]

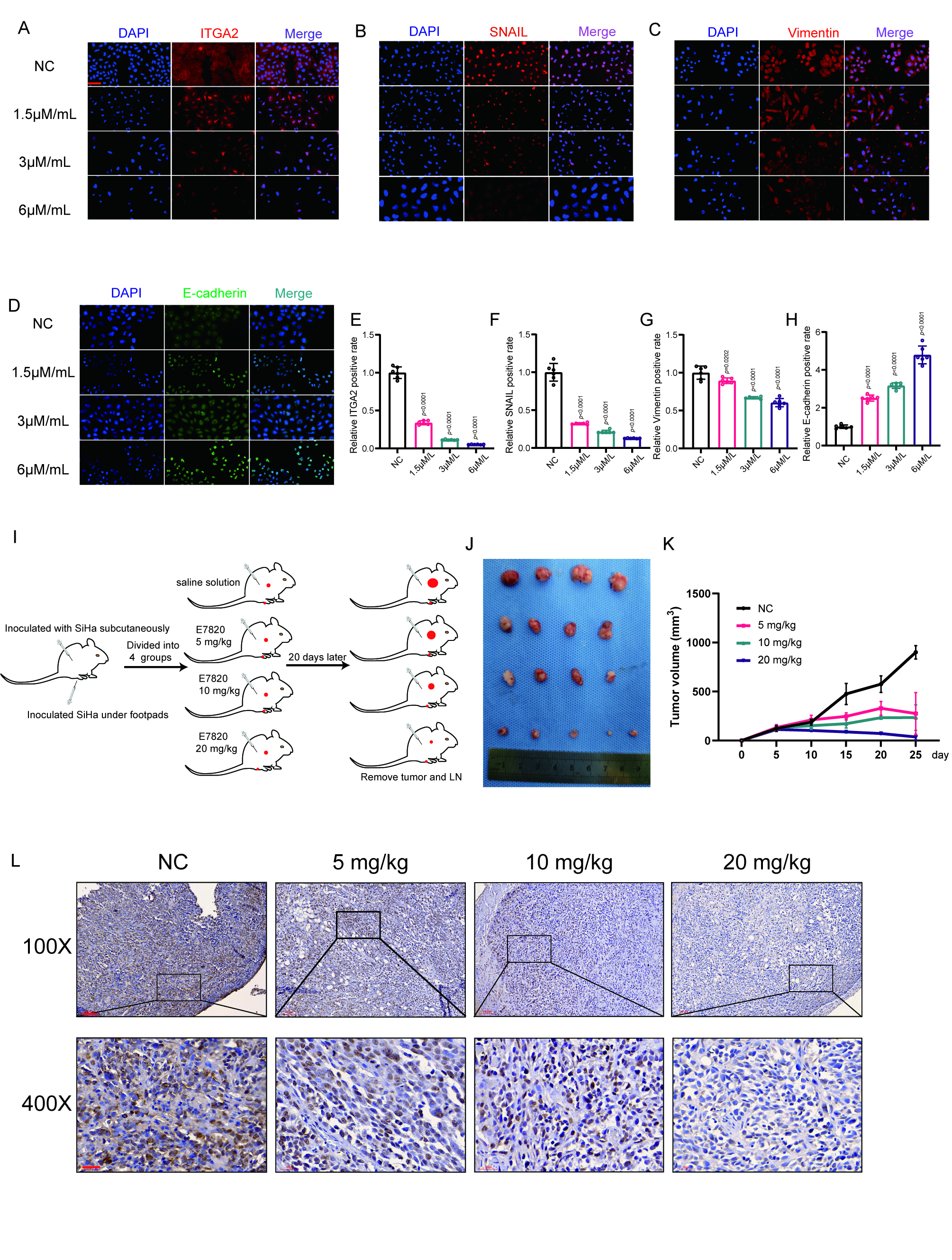

Supplement: Multimedia component 2 [file mmc2.zip › S4.tif]

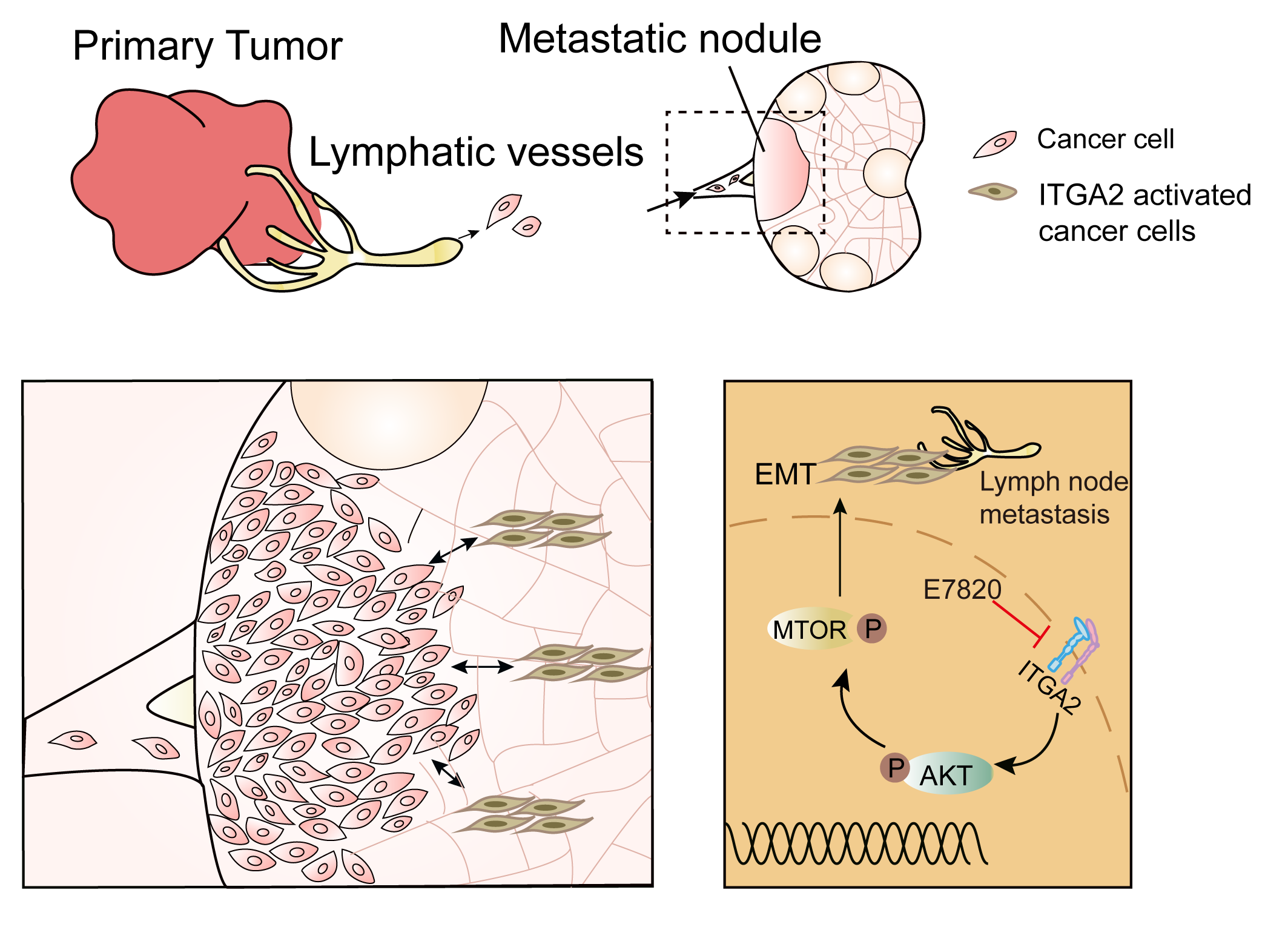

Supplement: Multimedia component 2 [file mmc2.zip › s5.tif]
